# Zinc and Potassium Fertilizer Synergizes Plant Nutrient Availability and Affects Growth, Yield, and Quality of Wheat Genotypes

**DOI:** 10.3390/plants12122241

**Published:** 2023-06-07

**Authors:** Aneela Bashir, Qudrat Ullah Khan, Ahmad Alem, Awatif A. Hendi, Umber Zaman, Shahid Ullah Khan, Khalil ur Rehman, Asghar Ali Khan, Ihsan Ullah, Yasir Anwar, Ehab A. Abdelrahman

**Affiliations:** 1 Department of Soil Sciences, Faculty of Agriculture, Gomal University, Dera Ismail Khan 29111, Pakistan; 2 Adult Critical Care & Emergency Consultant Emergency Department, King Saud Medical City, Riyadh 12746, Saudi Arabia; 3 Department of Physics, College of Science, Princess Nourah Bint Abdulrahman University, P.O. Box 84428, Riyadh 11671, Saudi Arabia; 4 Institute of Chemical Sciences, Gomal University, Dera Ismail Khan 29050, Pakistan; 5 Integrative Science Centre of Germplasm Creation in Western China (CHONGQING) Science City and Southwest University, College of Agronomy and Biotechnology, Southwest University, Chongqing 400715, China; 6 Department of Biochemistry, Women Medical and Dental College, Khyber Medical University, Peshawar 25100, Pakistan; 7 Department of Agronomy, Gomal University, Dera Ismail Khan 29111, Pakistan; 8 Department of Biological Sciences, Faculty of Science, King Abdulaziz University, Jeddah 21589, Saudi Arabia; 9 Department of Chemistry, College of Science, Imam Mohammad Ibn Saud Islamic University (IMSIU), Riyadh 11623, Saudi Arabia; 10 Chemistry Department, Faculty of Science, Benha University, Benha 13518, Egypt

**Keywords:** wheat, landrace, potassium, zinc, quality

## Abstract

The growth and productivity of wheat crops depend on the availability of essential nutrients such as zinc (Zn) and potassium (K_2_O), which play critical roles in the plant’s physiological and biochemical processes. This study aimed to investigate the synergizing effect of zinc and potassium fertilizers on uptake of both the nutrients, growth, yield, and quality of the Hashim-08 cultivar and local landrace, during the 2019–2020 growing season in Dera Ismail Khan, Pakistan. The experiment was designed using a split plot pattern in a randomized complete pattern, with main plots for the wheat cultivars and subplots for the fertilizer treatments. Results indicated that both cultivars responded positively to the fertilizer treatments, with the local landrace exhibiting maximum plant height and biological yield, and improved Hashim-08, showing increased agronomic parameters, including the number of tillers and grains and spike length. Application of Zn and K_2_O fertilizers significantly enhanced agronomic parameters, such as the number of grains per plant, spike length, thousand-grain weight, grain yield, harvest index, Zn uptake of grain, dry gluten content, and grain moisture content, while crude protein and grain potassium remained relatively unchanged. The soil’s Zn and K content dynamics were found to vary among treatments. In conclusion, the combined application of Zn and K_2_O fertilizers proved beneficial in improving the growth, yield, and quality of wheat crops, with the local landrace exhibiting lower grain yield but greater Zn uptake through fertilizer application. The study’s findings highlight that the local landrace showed good response to the growth and qualitative parameter when compared with the Hashim-08 cultivar. Additionally, the combined application of Zn and K showed a positive relation in terms of nutrient uptake and soil Zn and K content.

## 1. Introduction

Zinc is considered the most important micronutrient for many plant functions, including enhanced germination, growth regulation through enhanced enzyme reactions essential for photosynthesis, and the development of crops [1,2]. A deficiency of Zn is commonly observed in plants and human beings. In plants, it limits the productivity of crops and also lowers the nutritional quality of grains [3]. Wheat is inherently low in zinc content and high in phytate content which limits Zn bioavailability [4]. People of underdeveloped countries mainly consume wheat as their staple food and Zn deficiency is common in their diet [5,6].

Wheat (*Triticum aestivum*) is an important cereal crop providing food to an estimated 35% of the population of the world [7]. The productivity of wheat has increased over the years but due to the unavailability of micronutrients, the quality of wheat has declined. Some researchers have reported that zinc deficiency has been aggravated in wheat due to the adoption of improved varieties for obtaining higher yields [8,9,10]. A locally domesticated wheat, which has been adopted by farmers, developed over time, and adapted to the environmental condition of an area is called a landrace. Zinc content in the wheat grains is low due to various factors and, amongst these factors, the most common are higher soil CaCO_3_ content, soil alkalinity, high dosage of phosphoric fertilizer application, etc. Zinc shows various types of interaction in soil with other nutrients. Soil with high Ca, Fe, and P antagonizes their availability to plants. As phosphorus is a macronutrient and its application to plants is important for physiological and reproductive functions of crops, its regular and higher application dosages mask the availability of Zn. The interaction between zinc and phosphorus has been widely studied for different crops, however, the interaction between potassium and zinc in the presence of phosphorus-rich application under calcareous soil needs to be studied.

Potassium (K) is the third macronutrient required by plants for physiological and reproductive processes. There are a number of plant functions associated with potassium, including water and nutrient movement, enzyme activation, root growth, drought resistance, production of starch-rich grains, improving the quality of the grains, etc. Rehman et al. [11] reported that the antagonistic influence of phosphorus on zinc was lessened by increasing levels of potassium under rice–wheat cropping. In addition, the integrated application of K and Zn significantly enhanced the K uptake in maize [12]. The current study was designed to evaluate the potential traits of a local landrace for Zn and K uptake in comparison with improved Hashim-08. Additionally, the interactions between Zn and K in soil and plants were compared.

## 2. Material and Methods

A field experiment to investigate the response of a wheat variety and local landrace to Zn and K_2_O fertilizers was conducted at the Department of Soil Science, Gomal University, Dera Ismail Khan (Pakistan). The experimental design used was a randomized complete block with a split plot arrangement. Improved wheat variety Hashim-08 and a landrace (local name: Pehli farmi) were arranged by main plot, while the potassium and zinc levels i.e., control, K_2_O @ 60 kg ha^−1^, K_2_O @ 90 kg ha^−1^, Zn @ 3 kg ha^−1^, Zn @ 5 kg ha^−1^, Zn @ 3 kg ha^−1^ + K_2_O @ 60 kg ha^−1^, Zn @ 3 kg ha^−1^ + K_2_O @ 90 kg ha^−1^, Zn @ 5 kg ha^−1^ + K_2_O @ 60 kg ha^−1^, and Zn @ 5 kg ha^−1^ + K_2_O @ 90 kg ha^−1^ were arranged by subplot. Zinc sulfate (ZnSO_4_) and sulfate of potash (K_2_SO_4_) fertilizers were used as a source of Zn and K_2_O. Nitrogen and phosphorus were applied at the recommended rate of 120:90 kg ha^−1^ using urea and single super phosphate to all the plots under study.

### 2.1. Soil Analysis

A composite soil sample was analyzed for soil characteristics before the start of the experiment (Table 1). After the harvest of the wheat crop the soil samples were collected from each plot and analyzed for soil pH, electrical conductivity [13], soil organic matter [14], bulk density [15], total N, extractable P [16], exchangeable K [17], and soil Zn [18].

### 2.2. Grain Analysis

- Moisture Content:
  Moisture content in the wheat grains was determined in each sample by the method given by AACC [19] method No. 44-15.
- Crude Protein:
  Crude protein content in the grains was determined using the Kjeldhal method. After the determination of the percent nitrogen, the protein was calculated by multiplying it with a factor of 5.7.
- Dry Gluten:
  Dry gluten on a weight basis was determined gravimetrically and the calculation was carried out by the following formula [19].
  $$\begin{array}{c} Dry\;gluten=Weight\;of\;drygluten\times 100\times 100\;Exact\;weight\;of\;sample\\ \times {\left(100-Moisture\;content\right)}^{} \end{array}$$
- Zinc Uptake:
  After the determination of Zn using an atomic absorption spectrometer the uptake by the wheat grain was calculated by using the Chapman and Pratt [20] method. The following formula was used:
  $$Uptake\;of\;Zn({kg\;ha}^{-1})=\frac{Zn\%\times Dry\;matter\;(grain)\;{kg\;ha}^{-1}}{100}$$
- Potassium Uptake:
  Potassium concentration in the grain was determined using the flame photometer, while the uptake by the grain was calculated by using the following formula:
  $$Uptake\;of\;K({kg\;ha}^{-1})=\frac{K\;content\times Dry\;matter\;(grain)\;{kg\;ha}^{-1}}{100}$$

### 2.3. Agronomic Parameters

Wheat growth and yield parameters studied in the experiment included plant height (cm), number of spikes plant^−1^, spike length, thousand-grain weight (g), grain spike^−1^, grain yield (kg ha^−1^), biological yield (kg ha^−1^), and harvest index (%).

### 2.4. Statistical Analysis

Statistical analysis was carried out using Statistic 8.1 software for the determination of analysis of variance techniques and an LSD test at 5% was used to compare the differences among the treatment means [21].

## 3. Results

### 3.1. Agronomic Parameters of Wheat as Affected by the Application of Zinc and Potassium Fertilizers

#### 3.1.1. Plant Height

Plant height showed significant variation amongst the local landrace and Hashim-08. Taller plants were recorded in the local landrace, called Pehli farmi, when compared with the Hashim-08 cultivar (Table 2). However, the application of both potassium and zinc fertilizers showed an influence on plant stature. The tallest plants were found when the landrace Pehli farmi was grown on soil fertilized with Zn and K_2_O at 3 kg ha^−1^ and 90 kg ha^−1^. It was found that the landrace did not show a significant effect of the fertilizer treatment on the height of the plant when compared to the improved Hashim-08, which significantly responded to the fertilizer application. The tallest plants were found where fertilizer was applied at the maximum and the shortest in the treatment receiving no fertilizers.

#### 3.1.2. Number of Tillers per Plant

The number of tillers per plant was significantly affected by the application of fertilizer treatments in both varieties. The highest number of tillers was 20.36 in Hashim-08 where Zn and K_2_O were applied at 5 kg ha^−1^ and 90 kg ha^−1^. It is apparent from the research that the number of tillers per plant varied with the genotype of wheat, therefore significant variation amongst the cultivars was observed. Potassium enhanced the number of tillers per plant. The least number of tillers was 9.99 in the control of the landrace.

#### 3.1.3. Spike Length

Spike length is an important parameter that influences the productivity of a crop. The current experiment showed the longest spikes of 11.83 cm in Hashim-08 when treated with Zn and K_2_O fertilizer at the maximum level. It was statistically similar to the treatment where both Zn and K_2_O were applied at 3 kg ha^−1^–90 kg ha^−1^ and 5 kg ha^−1^–60 kg ha^−1^, respectively. The landrace exhibited a shorter spike length as compared with Hashim-08. The control of the landrace Pehli farmi exhibited a shortest spikes but a stronger response when the Zn and K_2_O fertilizers were applied in the experiments. In the soil fertilized with 5 kg ha^−1^ Zn and 90 kg ha^−1^ K_2_O the spike length of cultivar Hashim-08 increased 117% while that of the landrace Pehli farmi increased by 130 % compared to their controls.

#### 3.1.4. Number of Grains per Spike

Number of grains per spike showed great variation in the studied wheat cultivar and landrace. The results indicated that both potassium and zinc levels significantly affected the number of grains per spike. Although the landrace Pehli farmi gave the least number of grains in the control, its genetic potential may mean that, with an increasing dosage of K_2_O and Zn, the number of grains would be significantly enhanced. When the studied cultivar and the landrace were grown on maximum levels of Zn and K_2_O the cultivar grains per spike increased 119%, while the landrace increase was 115%, compared to their controls.

#### 3.1.5. Thousand-Grain Weight

Amongst the factors contributing to yield, thousand-grain weight is an important trait of wheat. The result of the current study indicated a significant effect of zinc and potassium fertilizer on the thousand-grain weight (Table 3). The highest thousand-grain weight was 45.44 g in Hashim-08 plots receiving Zn and K_2_O at 5 and 90 kg ha^−1^, respectively, which was statistically on par with Zn and K_2_O used at 5 and 60 kg ha^−1^. The least thousand-grain weight was of the landrace control.

#### 3.1.6. Grain Yield

The grain yield of wheat showed a significant difference between the two cultivars when grown under different levels of zinc and potassium fertilizer. The grain yield of 4544.25 kg ha^−1^ was the highest measured in the improved variety when applied with a maximum dosage of Zn and K_2_O fertilizer. The increasing level of both fertilizers significantly enhanced the grain yield in both varieties. The grain yield potential of the cultivars significantly varied. The landrace has the least potential to give greater grain yield. The lowest grain yield of 2992.5 kg ha^−1^ was recorded in the control where no Zn and K_2_O fertilizers were applied.

#### 3.1.7. Biological Yield

The results about the biological yield of wheat showed significant variation between the two cultivars and the increasing fertilizer levels. The highest biological yield of 11,659 kg ha^−1^ was found in the landrace cultivar when applied with Zn and K_2_O at 5 and 60 kg ha^−1^. It was statistically on par with the treatment of Zn and K_2_O at 5 and 90 kg ha^−1^ and 3 and 90 kg ha^−1^, respectively, and K_2_O only at 90 kg ha^−1^ in the same cultivar. The improved variety, due to its dwarf stature, had a significantly lower biological yield. The lowest biological yield was 9947 kg ha^−1^ of the Hashim-08 control.

#### 3.1.8. Harvest Index

Harvest index indicates the ratio between grain and biological yield. In the current study, the harvest index determined after the harvest of the crop indicated that Hashim-08 performed better in terms of grain yield with the highest harvest index of 43.14%. In terms of fertilizer application, it is evident that K_2_O significantly influenced the harvest index by enhancing the grain yield of Hashim-08. The landrace, due to greater biological mass, showed better vegetative growth but had the lowest harvest index. Amongst the fertilizer treatments, the lowest value of the harvest index was 28.56 for the control.

### 3.2. Qualitative Parameters of Wheat as Affected by the Application of Zinc and Potassium Fertilizers

#### 3.2.1. Grain Zn Uptake

The grain zinc uptake of wheat showed a significant effect of Zn and K_2_O fertilizer (*p* < 0.05). The data indicated that the Zn content of grain was enhanced by the application of Zn and K_2_O fertilizers (Table 4). The highest Zn was recorded in the grains of the landrace which yielded 32.62 mg kg^−1^. It was statistically on par with the rest of the treatments except the control. The data also indicated that the use of potassium fertilizer synergized with the Zn content in wheat grains.

#### 3.2.2. Grain K Uptake

The grain potassium uptake of wheat was non-significantly changed by the application of potassium and Zn fertilizer application. However, the highest value of 165.75 was recorded in the landrace where K only at 90 kg ha^−1^ was applied.

#### 3.2.3. Dry Gluten Content

The dry gluten content of wheat flour showed significant variation between the two cultivars. The highest dry gluten content was 12.80% in the control, whereas the lowest was 11.48% in the treatment where Zn and K_2_O were applied. The results revealed the treatments with only potassium and potassium in combination with Zn fertilizer reduced the gluten content.

#### 3.2.4. Crude Protein

The crude protein content of wheat was non-significantly affected by the use of fertilizers amongst the wheat cultivars. However, the fertilizer enhanced the crude protein content of grains.

#### 3.2.5. Grain Moisture Content

Grain moisture content was significantly changed by the application of Zn and K_2_O fertilizers in the two wheat cultivars. The highest moisture content was recorded in the improved Hashim-08 varieties, which yielded the highest value of 12.67% in the treatment receiving 90 kg ha^−1^ potassium only, which was statistically on par with the rest of the treatments used in this improved variety. The grain moisture content was the lowest in the landrace for different treatments of Zn and K_2_O fertilizer. The lowest moisture content of 10.03% was recorded in the control of the landrace.

#### 3.2.6. Soil Potassium Content

**Figure 1:**
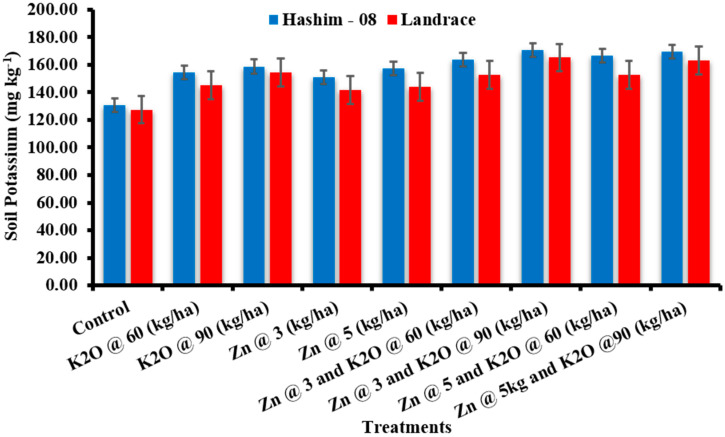
Soil potassium as affected by the zinc and potassium fertilizer.

Zinc and potassium fertilizers showed non-significant influence on the soil’s potassium content. However, the potassium and zinc fertilizers caused an increase in the soil K content (Figure 1).

#### 3.2.7. Soil Zinc Content

**Figure 2:**
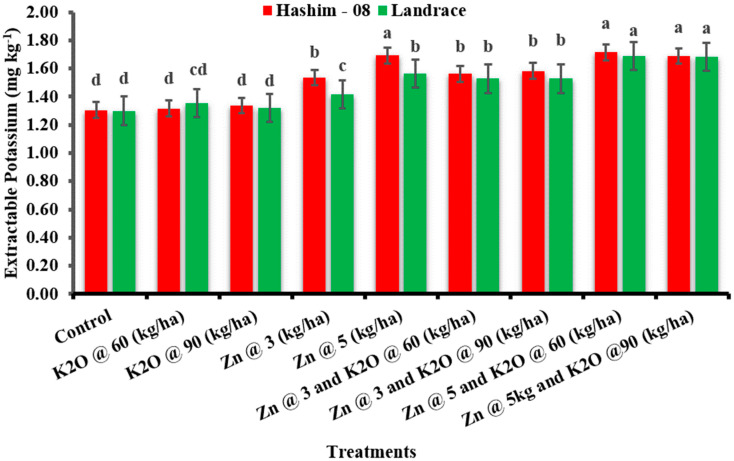
Soil zinc as affected by the zinc and potassium fertilizer.

Soil zinc content showed a significant effect of zinc and potassium fertilizer (Figure 2). The highest zinc content was recorded in the treatment of Zn 5 kg ha^−1^ + K_2_O 60 kg ha^−1^. It was statistically on par with the treatments of Zn 5 kg ha^−1^ + K_2_O 90 kg ha^−1^ and Zn 5 kg ha^−1^. The lowest soil Zn was recorded in the control.

## 4. Discussion

The growth and yield parameters in the current study showed greater variation between the improved variety and local landrace by application of Zn and potassium fertilizer. Aboyeji et al. [22] revealed that the application of Zn and K fertilizers significantly affected plant height. Other researchers have reported that Zn fertilizer has a role in various physiological processes, i.e., chlorophyll formation, activation of enzymes, stomatal regulation, etc., which increase the plant height [23,24,25]. Ali et al. [26] reported similar results for the number of tillers m^−2^ by the application of potassium fertilizers. Sadeghi et al. [27] reported that the application of zinc and magnesium did not affect the tillers of the crop and considered this trait to be controlled by genetic factors. Sher et al. [28] found longer spikes in the variety Fakhr–e–Sarhad by application of zinc sulfate. Additionally, Prajapati et al. [29] reported greater spike length when potassium and zinc were applied at 375 and 15 kg ha^−1^, respectively.

The application of potassium has been reported to increase the number of grains in wheat by improving nutrient uptake and enhancing nutrient mobilization and photosynthetic activity [30]. Aboyeji et al. [22] reported more grains per spike in a treatment of zinc both as a soil and foliar application. Al-Hiti and Al-Ubaidi [31] reported that the difference in the number of grains per spike in genotypes may be attributed to the difference in the genotypes’ efficiency to accumulate dry matter and then convert it to reproductive growth. Potassium has a significant role in the growth and cell division of plants, as it enhances the water use efficiency of the crop and helps in the synthesis of protein and metabolites. Moreover, potassium is known to regulate the synthesis, conversion, and distribution of metabolites, which eventually result in the enhancement of yield [32]. Potassium is an essential macronutrient responsible for grain development and showed a favorable effect on grain yield when applied at a higher level [33]. Brhane et al. [34] revealed that potassium applied at 30, 60, and 90 kg ha^−1^ significantly enhanced the grain yield of wheat, however, 60 kg ha^−1^ proved to be the most economical treatment. In addition, zinc has a significant role in enhancing yield when applied at successively increasing levels of 3, 6, and 9 kg ha^−1^ with an increase in grain yield of 13, 24, and 30%, respectively [33]. Firdous et al. [35] found a higher yield of wheat by the application of zinc as a basal and foliar dose.

Al-Taher and Al-Naser [36] found a significant increase in biological yield through the application of potassium fertilizers. Khan et al. found a higher biological yield of wheat by increasing the potassium level up to 60 kg ha^−1^ while a further increment in K level decreased biological yield. Similarly, results for the harvest index of wheat are reported by Arif et al. (2017), and they observed an increase in harvest index by the application of potassium and Zn at 375 kg ha^−1^ and 5 kg ha^−1^, respectively. Similar results have been reported by Brhane et al. [34] in which the harvest index was significantly increased by the application of K. The highest was recorded for 90 kg K_2_O ha^−1^. Firdous et al. [35] found greater Zn content in wheat grains in a treatment where both basal and foliar dosages of Zn were applied. Jat et al. [33] found zinc uptake in wheat grain of 81.2 g ha^−1^ under 9 kg Zn ha^−1^ which resulted from a 54% increase in Zn uptake in grain. Zhang et al. [37] reported that Zn content in grain increased by 58%.

Qualitative parameters including Zn and K uptake, gluten content, crude protein, and moisture content in wheat grains were studied during the experiment. Roshani and Narayanasamy [38] reported a similar result in wheat grains and further depicted a higher uptake of potassium by cereal crops before maximum maturity is reached. Potassium content decreases as the grain fully matures and K is lost from the plant in most cereal crops. Hafeez et al. [39] observed significant variation in the gluten content of wheat grains and considered the type of wheat varieties responsible for it and soil-applied Zn. In their research, a higher gluten content was recorded in the control and application of Zn fertilizers significantly reduced the gluten content in one of the wheat varieties under study. Mahmood (2004) depicted that different wheat varieties have variations in crude protein content, ranging from 9.71% to 15.42%. Turk et al. [40] revealed that crude protein synthesis mainly depends on the dry matter yield of the plant, which changes with plant species. As the fertilizer enhances the dry matter production, the crude protein content is increased. Moisture content in wheat grains increases with the increase in the level of NPK fertilizers [41].

Soil potassium and zinc content during the study showed a significant effect of treatments. The increase in soil potassium has been reported by Tariq et al. [42], who observed that the increment of K in soil may be due to the transformation of non-exchangeable K to exchangeable K. A similar result has been reported by Jat et al. [33] who reported that analyzed Zn fractions after crop harvest indicate that K levels did not significantly affect the soil Zn fraction, however, different soil Zn fractions were significantly influenced by the Zn levels applied as an amendment. Some researchers have fortified Zn in combination with N, P, and K applied as foliar applications [43].

## 5. Conclusions

The nub of present scientific study revealed that landraces are the best source material for the investigation and exploration of the various traits under study. Inclusion of a landrace in any wheat hybridization program can show a marked effect on wheat yield and yield-related attributes. Moreover, integration of zinc and potassium also exhibited a significant synergetic relation in soil and plants by influencing the uptake of both nutrients. In this study it has been found that wise and judicious use of zinc and potassium may amplify wheat yield and sustained quality. There is need to carry out further research on the evolution of landraces into improved varieties by selecting and crossing the promising traits specifically linked to plant nutrition.

## Figures and Tables

**Table 1:** Characterization of soil prior to the experiment.

Soil Parameters	Soil Texture	Soil pH	Soil EC (mScm^−1^)	Bulk Density (gcm^−3^)	Organic Matter (%)	Total Nitrogen (%)	Extractable Phosphorus (mg kg^−1^)	Extractable Potassium (mg kg^−1^)	Soil Zinc Content (mg kg^−1^)
**Value**	Silty Clay	7.94	1.45	1.35	0.58	0.035	6.11	141	1.54

**Table 2:** Growth parameters of wheat as affected by the zinc and potassium fertilizers.

Treatment	Plant Height (cm)		No. of Tillers per Plant		Spike Length (cm)		No. of Grains per Spike	
	Hashim-08	Landrace	Hashim-08	Landrace	Hashim-08	Landrace	Hashim-08	Landrace
Control	86.12 d	107.88 a	14.07 de	9.99 g	10.07 efg	8.05 h	41.20 g	31.16 k
K_2_O @ 60 kg ha^−1^	87.29 d	109.51 a	15.13 cd	11.50 fg	10.16 efg	8.70 h	43.67 f	32.90 ijk
K_2_O @ 90 kg ha^−1^	90.93 bc	110.85 a	17.08 bc	12.15 ef	10.77 bcde	8.77 h	46.90 bcd	34.59 hi
Zn @ 3 kg ha^−1^	86.04 d	110.28 a	15.20 cd	10.81 fg	10.48 cdef	9.58 g	44.71 ef	32.36 jk
Zn @ 5 kg ha^−1^	88.87 bcd	109.19 a	15.45 cd	10.94 fg	11.09 abcd	10.07 efg	45.84 de	33.97 hij
Zn @ 3 kg ha^−1^ + K_2_O @ 60 kg ha^−1^	90.96 bc	110.57 a	19.02 ab	11.67 fg	11.23 abc	9.81 fg	46.58 cde	32.81 ijk
Zn @ 3 kg ha^−1^ + K_2_O @ 90 kg ha^−1^	92.06 b	111.50 a	19.34 a	12.33 ef	11.64 a	10.38 def	48.52 ab	34.39 hi
Zn @ 5 kg ha^−1^ + K_2_O @ 60 kg ha^−1^	89.64 bcd	110.74 a	17.03 bc	11.73 fg	11.38 ab	10.00 efg	47.93 abc	33.29 ij
Zn @ 5 kg ha^−1^ + K_2_O @ 90 kg ha^−1^	90.99 bc	111.04 a	20.36 a	12.46 ef	11.83 a	10.51 cdef	49.01 a	35.84 h
LSD _Interaction_	3.8658		2.0611		0.7583		1.9355	

Means with dissimilar letters in a column are significantly different at 5% level of significance.

**Table 3:** Yield parameters of wheat as affected by the zinc and potassium fertilizers.

Treatment	Thousand Grain Weight (g)		Grain Yield (kg ha^−1^)		Biological Yield (kg ha^−1^)		Harvest Index	
	Hashim-08	Landrace	Hashim-08	Landrace	Hashim-08	Landrace	Hashim-08	Landrace
Control	41.20 jk	40.55 k	4119.75 e	2992.50 j	9947.25 f	10,492.50 e	41.43 bcd	28.56 h
K_2_O @ 60 kg ha^−1^	42.98 efgh	41.64 fghi	4297.75 cd	3345.50 i	10,072.75 f	10,916.50 cd	42.67 a	30.66 efg
K_2_O @ 90 kg ha^−1^	44.40 abcd	42.54 fghi	4440.00 ab	3416.50 hi	10,440.00 e	11,446.50 a	42.53 abc	29.84 gh
Zn @ 3 kg ha^−1^	42.48 fghi	42.57 fghi	4247.50 d	3387.00 i	10,247.50 ef	11,374.75 ab	41.44 bcd	29.77 gh
Zn @ 5 kg ha^−1^	42.13 ghij	41.96 hij	4213.00 de	3446.50 hi	10,213.00 ef	11,095.50 bc	41.25 d	31.12 ef
Zn @ 3 kg ha^−1^ + K_2_O @ 60 kg ha^−1^	44.08 bcde	43.39 cdef	4407.75 bc	3504.25 gh	10,407.75 e	11,504.25 a	42.35 abcd	30.46 efg
Zn @ 3 kg ha^−1^ + K_2_O @ 90 kg ha^−1^	44.44 abc	44.06 bcde	4443.75 ab	3617.00 fg	10,443.75 e	11,617.00 a	42.55 abc	31.13 ef
Zn @ 5 kg ha^−1^ + K_2_O @ 60 kg ha^−1^	44.65 ab	43.01 efgh	4464.50 ab	3659.25 f	10,464.50 e	11,659.25 a	42.66 ab	31.38 e
Zn @ 5 kg ha^−1^ + K_2_O @ 90 kg ha^−1^	45.44 a	43.23 defg	4544.25 a	3621.50 f	10,533.50 de	11,621.50 a	43.14 a	31.16 ef
LSD _Interaction_	1.2483		113.94		330.04		1.2177	

Means with dissimilar letters in a column are significantly different at 5% level of significance.

**Table 4:** Qualitative parameters of wheat as affected by the zinc and potassium fertilizers.

Treatment	Grain Zn Uptake (mg kg^−1^)		Grain K Uptake (kg ha^−1^)		Dry Gluten (%)		Crude Protein (%)		Grain Moisture Content (%)	
	Hashim-08	Landrace	Hashim-08	Landrace	Hashim-08	Landrace	Hashim-08	Landrace	Hashim-08	Landrace
Control	26.20 ab	23.96 b	128.50 ^NS^	141.00	12.80 a	12.55 ab	10.43 ^NS^	10.84	12.66 a	10.03 d
K_2_O @ 60 kg ha^−1^	29.84 ab	26.97 ab	143.75	148.75	11.76 ab	11.77 ab	11.09	11.20	12.37 a	10.62 c
K_2_O @ 90 kg ha^−1^	25.27 ab	29.66 ab	160.00	165.75	11.87 ab	11.90 ab	11.25	11.55	12.67 a	10.78 c
Zn @ 3 kg ha^−1^	31.02 ab	29.01 ab	136.00	140.00	12.44 ab	12.46 ab	10.98	10.99	12.41 a	11.63 b
Zn @ 5 kg ha^−1^	28.00 ab	25.60 ab	138.50	147.25	12.60 ab	12.02 ab	11.00	10.98	12.58 a	11.51 b
Zn @ 3 kg ha^−1^ + K_2_O @ 60 kg ha^−1^	31.66 ab	25.45 ab	150.75	153.75	11.61 b	12.65 ab	11.19	11.07	12.46 a	11.68 b
Zn @ 3 kg ha^−1^ + K_2_O @ 90 kg ha^−1^	28.51 ab	26.49 ab	161.75	164.25	11.93 ab	11.80 ab	11.48	11.04	12.72 a	11.63 b
Zn @ 5 kg ha^−1^ + K_2_O @ 60 kg ha^−1^	29.59 ab	32.35 a	147.25	152.50	12.42 ab	11.48 b	11.17	11.41	12.47 a	11.50 b
Zn @ 5 kg ha^−1^ + K_2_O @ 90 kg ha^−1^	31.18 ab	32.62 a	155.50	161.25	11.84 ab	12.23 ab	11.41	11.27	12.66 a	11.44 b
LSD _Interaction_	7.31		NS		1.1759		NS		0.5173	

Means with different letters in a column are different significantly at 5% probability.

## Data Availability

Not applicable.
